# Erratum for Cai et al., “Systematic Microbiome Dysbiosis Is Associated with IgA Nephropathy”

**DOI:** 10.1128/spectrum.02686-23

**Published:** 2023-09-29

**Authors:** Fengtao Cai, Chenfen Zhou, Na Jiao, Xinling Liang, Zhiming Ye, Wei Chen, Qiongqiong Yang, Hui Peng, Ying Tang, Chaoqun Niu, Guoping Zhao, Zefeng Wang, Guoqing Zhang, Xueqing Yu

## ERRATUM

Volume 11, no. 3, e05202-22, 2023, https://doi.org/10.1128/spectrum.05202-22. Page 1: The corresponding authors should be as given in this Erratum.

