# Supplementary material for: Systematic Microbiome Dysbiosis Is Associated with IgA Nephropathy
Source: Microbiol Spectr. 2023 May 25;11(3):e05202-22. doi: 10.1128/spectrum.05202-22 (PMC10269816; doi:10.1128/spectrum.05202-22)
Supplement: Supplemental file 1 — Supplemental material. Download spectrum.05202-22-s0001.pdf, PDF file, 1.2 MB [file spectrum.05202-22-s0001.pdf]

TABLE S1 Clinical characteristics of IgAN patients in the discovery and validation cohort[a]

**Clinical indices**	**Discovery cohort (n=234)**		***P*-value**	**Validation cohort (n=65)**		***P*-value**
	**Advanced (n=98)**	**Early (n=136)**		**Advanced (n=21)**	**Early (n=44)**	
Age (years)	42.02±9.37	35.47±9.34	3.02E-07	40.67±10.16	36.82±8.55	0.19
Gender (female)	47 (47.96%)	79 (58.09%)	0.16	13 (61.9%)	21 (47.73%)	0.42
BMI (kg/m^2)	22.35±3.03	22.53±3.36	0.88	21.77±5.89	21.66±5.7	0.89
Creatinine (umol/L)	258.09±249.79	81.95±19.14	1.71E-36	167.56±68.62	85.99±28.12	2.40E-11
Urea (mmol/L)	11.32±6.93	5.27±1.38	1.19E-24	8.88±2.64	5.74±1.42	3.63E-08
eGFR (mL/min/1.73m^2)	35.09±17.17	92.68±18.78	6.97E-39	42.42±13.95	94.24±20.97	3.29E-17
Gd-IgA1/IgA1	0.93±0.66	0.8±0.41	0.50	0.77±0.44	0.8±0.51	0.97
IgA1 (ug/mL)	822.13±407.16	819.04±351.67	0.75	858.67±312.17	845.93±302.76	0.86
Gd-IgA1 (ug/mL)	6.64±4.64	5.85±2.71	0.53	5.84±2.72	6.02±3.09	0.91

[a]Continuous variables were expressed as means ± standard deviations and compared with Wilcoxon rank sum test; Categorical variables were expressed as percentages and compared with Chi-square test; BMI, body mass index; eGFR, estimated glomerular filtration rate; Gd-IgA1, galactose-deficient immunoglobulin A 1.

TABLE S2 Alpha diversity indices for IgAN patients *vs.* HCs[a]

Alpha diversity indices	Oral			Pharynx			Gut			Urine		
	IgAN (n=239)	HCs (n=124)	*P*-value	IgAN (n=242)	HCs (n=126)	*P*-value	IgAN (n=207)	HCs (n=112)	*P*-value	IgAN (n=186)	HCs (n=97)	*P*-value
Chao Index	239.27 ± 74.91	233.39 ± 87.41	0.39	206.95 ± 59.77	217.73 ± 67.86	0.0986	156.28 ± 60.75	169.35 ± 53.41	**0.0440***	150.67 ± 160.67	143.29 ± 132.68	0.6666
Simpson Index	0.87 ± 0.12	0.85 ± 0.12	**0.0142***	0.92 ±0.07	0.92 ±0.09	0.6347	0.92 ± 0.07	0.94 ± 0.04	**0.0095***	0.69 ± 0.21	0.73 ±0.2	0.0947
Shannon Index	3.49 ± 0.79	3.28 ± 0.82	**0.0198***	3.7 ±0.53	3.71 ±0.6	0.3789	3.54 ± 0.63	3.75 ± 0.5	**0.0034***	2.14 ± 0.99	2.27 ± 0.93	0.1722
ACE Index	238.91 ± 74.76	233.18 ± 87.1	0.3929	206.11 ± 59.22	217.27 ± 67.48	0.0999	156.41 ± 60.66	169.42 ± 53.28	**0.0440***	151.13 ± 160.51	143.51 ± 132.62	0.6683
Good's Coverage Index	1 ±0	1 ±0	0.1272	1 ±0	1 ±0	0.14	1 ±0	1 ±0	0.3199	1 ±0	1 ±0	0.3787

[a]Alpha diversity indices were expressed as means ± standard deviations and compared with Kruskal-Wallis test.*, $P < 0.05$.

TABLE S3 Microbial features of random forest models for IgAN *vs*. HCs

**Features**	**Importance**	**Niches**
Capnocytophaga	16.22829299	Oral
Bergeyella	9.90651681	Oral
Comamonas	9.611221803	Oral
Stomatobaculum	7.937609932	Oral
Pseudopropionibacterium	4.620556703	Oral
Oribacterium	4.214752395	Oral
Megasphaera	3.711087756	Oral
Gracilibacteria	3.666631714	Oral
Johnsonella	3.29241326	Oral
Cardiobacterium	2.865976451	Oral
Comamonas	12.12723802	Pharynx
Stomatobaculum	10.27368483	Pharynx
Delftia	8.431094637	Pharynx
Mogibacterium	8.420040069	Pharynx
Capnocytophaga	6.675234242	Pharynx
Alloprevotella	6.261374916	Pharynx
Bergeyella	5.166384098	Pharynx
Streptococcus	4.203681229	Pharynx
Gracilibacteria	4.145622121	Pharynx
Veillonella	4.058548746	Pharynx
Actinomyces	3.89153579	Pharynx
Rothia	3.684683201	Pharynx
F0332	3.232208143	Pharynx
Olsenella	3.14998443	Pharynx
Erysipelatoclostridium	8.912613226	Gut
Ruminococcus	6.797324169	Gut
Romboutsia	6.18271165	Gut
CAG-56	5.316319603	Gut
Allisonella	5.125618313	Gut
Coprococcus	4.809675708	Gut
Butyricicoccus	4.64259058	Gut
Monoglobus	4.568207749	Gut
Veillonella	4.034568773	Gut
Streptococcus	3.725710441	Gut
Candidatus_Soleaferrea	3.02872443	Gut
Clostridium_sensu_stricto_1	2.7308402	Gut
Aeromonas	15.60054839	Urine
Delftia	8.25979041	Urine
Allorhizobium-Neorhizobium-Pararhizobium-Rhizobium	7.130486461	Urine
Streptococcus	6.199831052	Urine
Finegoldia	5.966340803	Urine
Stenotrophomonas	5.754501135	Urine

Pseudomonas	4.577861661	Urine
Brevundimonas	4.271557335	Urine
Vibrionimonas	4.122466213	Urine
Ralstonia	3.595730797	Urine
Enterobacter	3.481829289	Urine
Peptoniphilus	3.272062178	Urine
Anaerococcus	3.136566391	Urine

TABLE S4 Microbial features of random forest models for early *vs.* advanced patients

**Features**	**Importance**	**Niches**
Bergeyella	6.2451637	Oral
Comamonas	5.951956	Oral
Gemella	5.1027366	Oral
Granulicatella	4.5564446	Oral
Gracilibacteria	4.3088531	Oral
Capnocytophaga	4.1716156	Oral
Veillonella	3.5694884	Oral
Actinomyces	3.5520921	Oral
Bacteroides	3.3283145	Oral
Bergeyella	14.260002	Pharynx
Comamonas	10.560553	Pharynx
Veillonella	8.4947074	Pharynx
Gracilibacteria	7.5920719	Pharynx
Capnocytophaga	6.5872686	Pharynx
Selenomonas	5.3887077	Pharynx
Candidatus_Saccharimonas	4.9720675	Pharynx
Defluviitaleaceae_UCG-011	4.6599411	Pharynx
Mogibacterium	3.6435606	Pharynx
F0058	3.1441735	Pharynx
Solobacterium	3.0339758	Pharynx
[Eubacterium]_yurii_group	2.9879354	Pharynx
[Ruminococcus]_torques_group	5.7099263	Gut
Flavonifractor	5.1764982	Gut
Allisonella	5.0795856	Gut
Raoultibacter	5.0183582	Gut
Rothia	4.1762447	Gut
Dialister	3.7597811	Gut
Bacteroides	3.7450585	Gut
Actinomyces	12.90708	Urine
Vibrionimonas	6.1270564	Urine
Delftia	5.7630911	Urine
Rhodococcus	3.6107918	Urine
Aeromonas	3.3846179	Urine

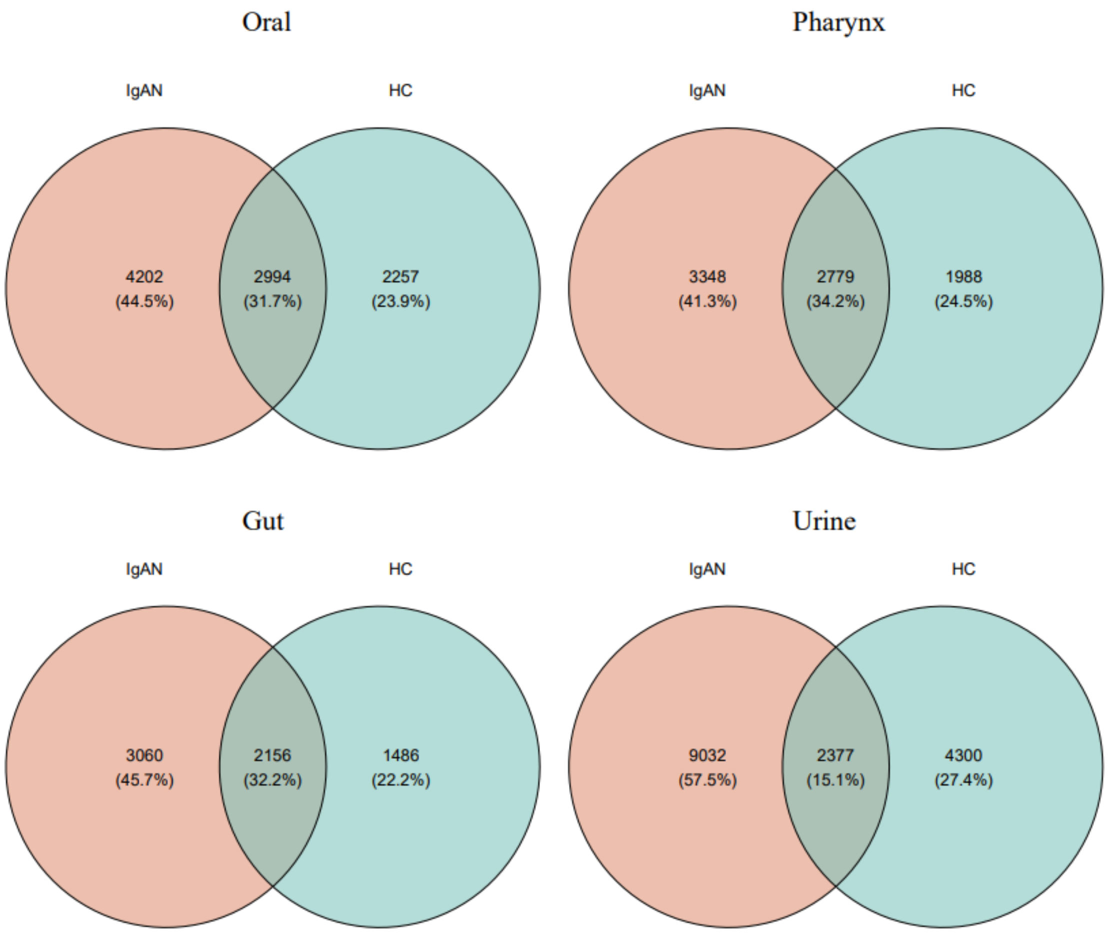

FIG S1 The Venn diagrams showed the shared and unique amplicon sequence variants (ASVs) between IgAN patients and healthy controls (HCs) in each niche.

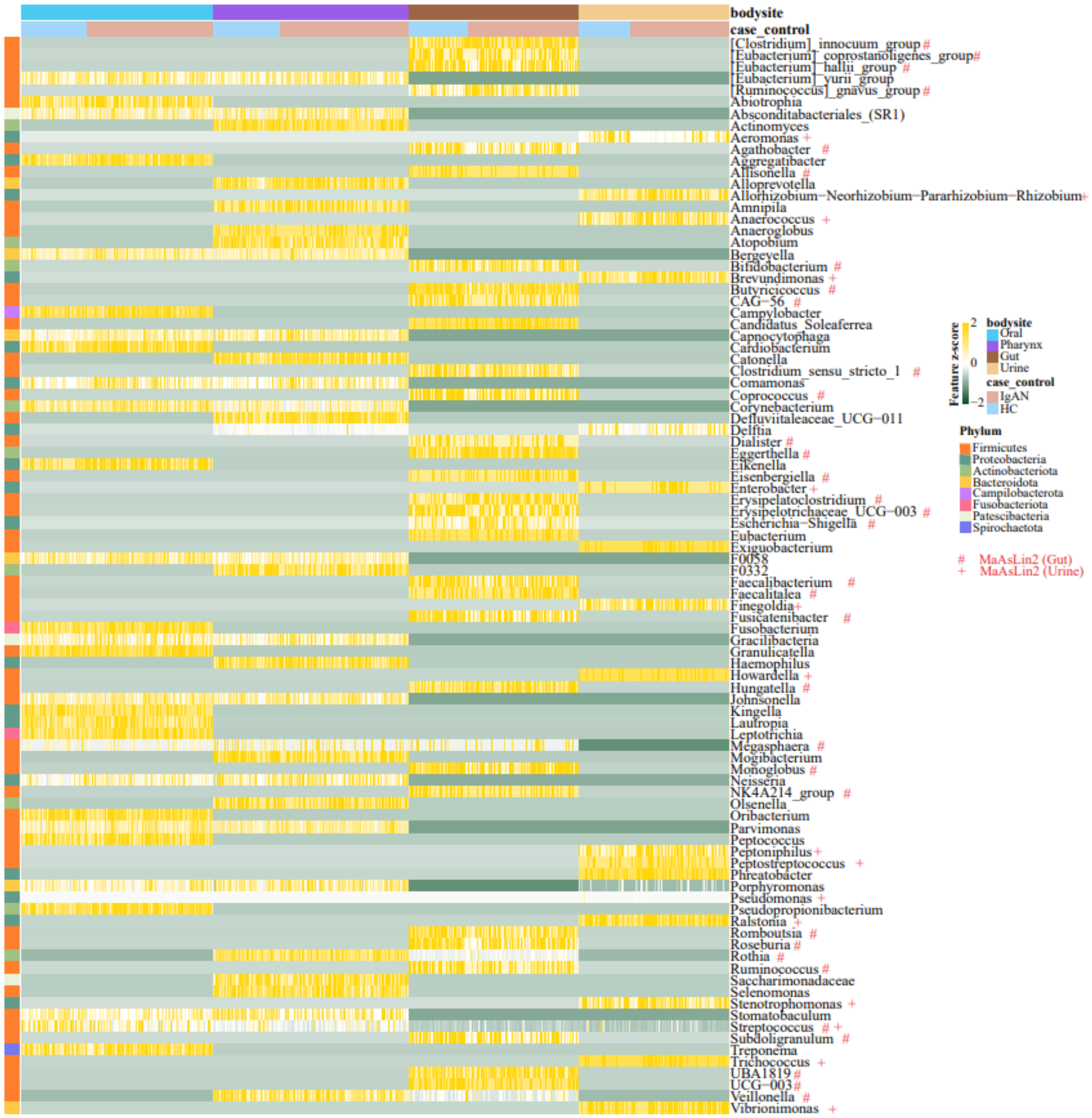

FIG S2 Differential microbial genera from each body site between IgAN patients and controls. Genera with different abundances were identified by Wilcoxon rank sum test and *P* values were further adjusted by FDR method in R, where the results of false discovery rate (FDR) *P* < 0.2 were shown in the heat map. The feature z-score was calculated on the log value of relative abundance (to avoid infinite values from the logarithm, a pseudo-count of 1e-06 was added to all values). Colors of body sites, groups and phylum were shown in the color bar. "#" showed bacterial taxa with distinct relative abundances between groups after adjusting for the gender using MaAsLin2 in gut samples; '+' showed bacterial taxa with distinct relative abundances between groups after adjusting for the BMI using MaAsLin2 in urine samples. MaAsLin, Microbiome Multivariable Associations with Linear Models.

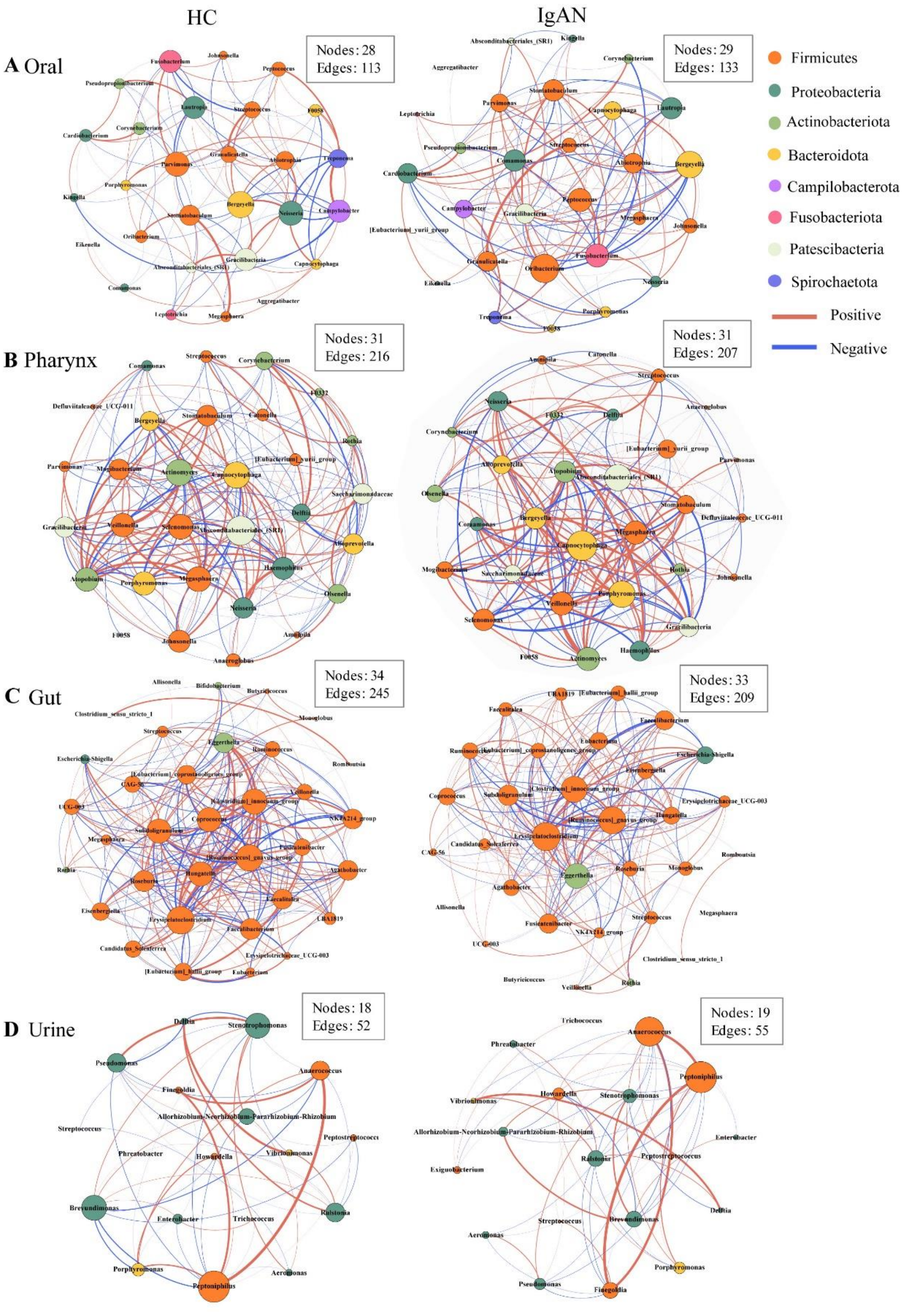

FIG S3 Co-abundance associations among differential microbial genera in IgAN patients and controls. (A-D) Co-abundance networks for oral, pharynx, gut and urine microbes, respectively. The associations among the differential genera were quantified by the SparCC methods, where the absolute value of correlation coefficient >= 0.2 and

FDR $P < 0.05$ were shown in the networks. The edge widths were proportional to the correlation strength. The orange edges showed the positive correlations and blue ones showed the negative correlations. The node sizes were proportional to the degree and the colors of nodes indicated genus from the phylum as shown in the color bar. The number of nodes and edges were shown in the upper right rectangle of each network. SparCC, Sparse Correlations for Compositional data.

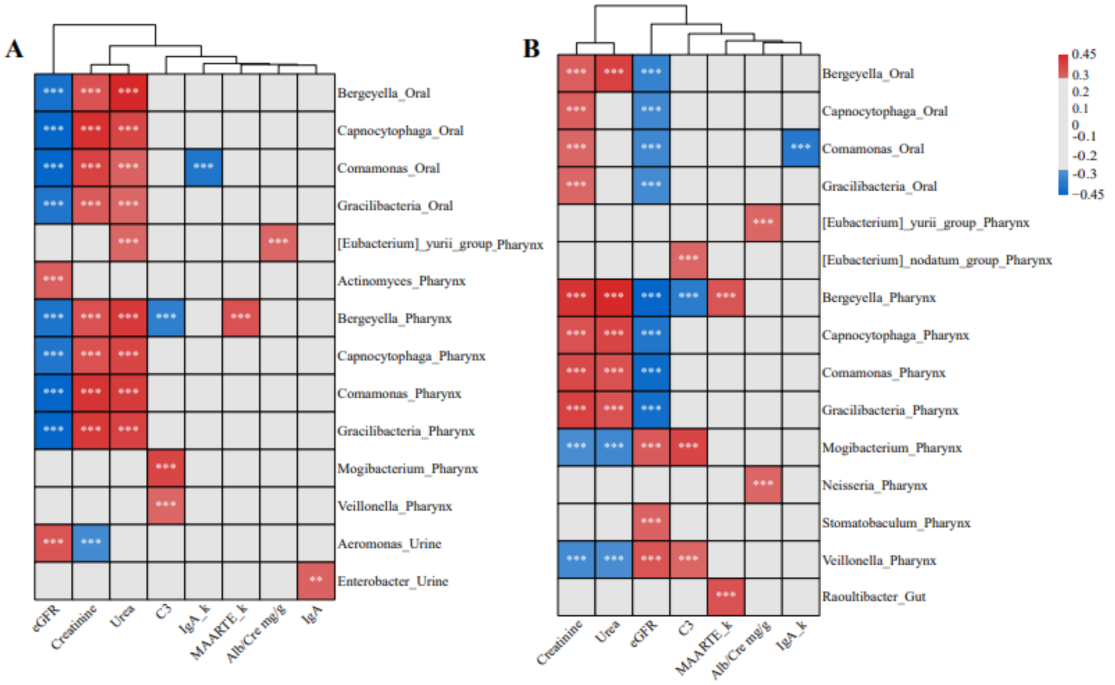

FIG S4 Associations between differential microbial genera and clinical indices of IgAN. (A) Associations between clinical parameters and differential microbial genera from IgAN patients against controls. (B) Associations between clinical parameters and differential microbial genera from early against advanced IgAN patients. The associations were calculated by Spearman's rank correlation method in R, where the absolute value of correlation coefficient >= 0.3 and FDR $P < 0.1$ were shown in the heat map. The red rectangle showed the positive association and the blue one showed the negative association. While the grey rectangle indicated that the absolute value of correlation coefficient was less than 0.3. *, FDR $P < 0.1$; **, FDR $P < 0.05$; ***, FDR $P < 0.01$. Alb/Cre mg/g, albumin/creatinine mg/g; eGFR, estimated glomerular filtration rate; IgA, immunoglobulin A; IgA_k, immunoglobulin A comes from kidney biopsy results; MAARTE_k, multifocal atrophic area of renal tubular epithelium comes from kidney biopsy results.

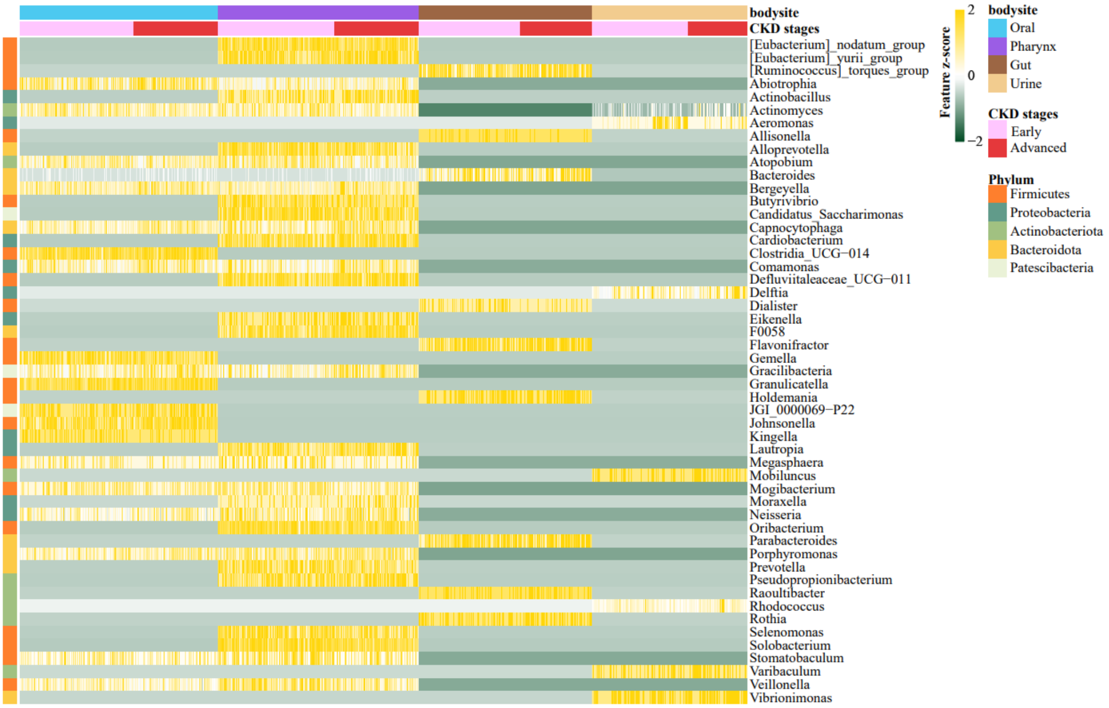

FIG S5 Differential microbial genera from each body site between early and advanced IgAN patients. Genera with different abundances were identified by Wilcoxon rank sum test and *P* values were further adjusted by FDR method in R, where the results of FDR $P < 0.2$ were shown in the heat map. The feature z-score was calculated on the log value of relative abundance (to avoid infinite values from the logarithm, a pseudo-count of 1e-06 was added to all values). Colors of body sites, CKD stages and phylum were shown in the color bar.
